# The periplasmic and cytoplasmic faces of septal protein SepJ from filamentous cyanobacteria

**DOI:** 10.1128/jb.00488-24

**Published:** 2025-03-31

**Authors:** Enrique Flores

**Affiliations:** 1 Instituto de Bioquímica Vegetal y Fotosíntesis, CSIC and Universidad de Sevilla 16778, Seville, Spain; Queen Mary University of London, London, United Kingdom

**Keywords:** cyanobacteria, septal junctions, intercellular communication, multicellularity

## Abstract

Filamentous, N_2_-fixing, heterocyst-forming cyanobacteria grow as chains of cells in which intercellular transfer of regulators and metabolites takes place, allowing them to behave as multicellular organisms. Intercellular transfer occurs by diffusion through septal junctions. In the model heterocyst-forming cyanobacterium *Anabaena* sp. strain PCC 7120, some identified septal proteins, including FraC and FraD, are directly involved in the formation of junctions that have been visualized by cryo-electron tomography, whereas the role of the key septal protein SepJ remains elusive. SepJ can form tetramers and contains coiled-coil, linker, and integral membrane (permease) domains. Using AlphaFold 3, a SepJ tetramer is predicted to have a quaternary structure in which the coiled-coil domain traverses the cytoplasmic membrane through a cavity formed between the four permease domains. Part of the coiled-coil domain is thus located in the septal periplasm, where it can interact with peptidoglycan. This possible SepJ structure can be widespread in filamentous cyanobacteria and explains known properties of SepJ. Structures of SepJ with other septal proteins including SjcF1, SepI, and SepT could also be predicted consistent with their previously described interactions. A possible interaction of the SepJ coiled-coil domain with the catalytic domain of cell wall amidase AmiC1, which would be relevant to prevent filament fragmentation in *Anabaena*, is also discussed. The renewed view of SepJ presented here offers a molecular basis for understanding the key role of this protein in filament formation and intercellular communication.

## INTRODUCTION

Heterocyst-forming cyanobacteria grow as filaments made of tens to hundreds of cells in which, under nitrogen-poor conditions, two types of cells can be found: vegetative cells that fix CO_2_ through oxygenic photosynthesis and heterocysts specialized for N_2_ fixation (1, 2). Differentiation of a vegetative cell into a heterocyst involves a complex program of gene expression that is activated upon nitrogen step-down (3, 4). The pattern of heterocysts in a filament, typically one heterocyst separated by 10–15 vegetative cells, is mainly established by inhibition of the differentiation of cells adjacent to differentiating or mature heterocysts; this is accomplished by inhibitory morphogens related to the *patS* and *hetN* genes that are produced by (pro)heterocysts and reach several cells away in their neighborhood (5–7). In the diazotrophic filament, heterocysts provide vegetative cells with fixed nitrogen, and vegetative cells provide heterocysts with reduced carbon; metabolites that are exchanged include glutamine, glutamate, sucrose, dipeptide β-aspartyl arginine, and others (reviewed in Herrero et al. [2]). Thus, intercellular molecular exchange is key to the diazotrophic physiology of heterocyst-forming cyanobacteria, whereas it also plays a role in the biology of these organisms when grown in the presence of combined nitrogen (8).

Recent work in this field, summarized below, has identified proteins and structures that are important for making long filaments and for intercellular molecular exchange. However, the role of a key protein, SepJ, remains elusive. Here, I present the possible structure of SepJ multimeric complexes as predicted by AlphaFold 3 (9). The predicted structure clarifies our view of SepJ permitting to understand previously obtained experimental results and to propose hypotheses on its role in the cyanobacterial filament.

## FILAMENT STRUCTURE AND INTERCELLULAR COMMUNICATION

Cyanobacteria are diderm bacteria (10). However, in filamentous cyanobacteria, the outer membrane does not enter the septum between adjacent cells, whereas each cell is surrounded by its cytoplasmic membrane (CM) and peptidoglycan (PG) layer (11). As measured in the heterocyst-forming cyanobacterium *Anabaena* sp. strain PCC 7120 (also known as *Nostoc* sp., hereafter *Anabaena*), the PG appears thicker in the intercellular septa (thickness, about 26–27 nm) than in the periphery of the cells (thickness, about 14 nm), likely reflecting juxtaposition of the PG layers of adjacent cells (10–12). Because murein sacculi corresponding to several cell units in the filament can be isolated, the PG layers of adjacent cells appear to be covalently linked (12). The septal PG can indeed be seen by transmission electron microscopy (TEM) as “septal disks”, and in these disks, holes about 20 nm in diameter that traverse the PG have been observed and termed nanopores (13).

In the cyanobacterial filaments, intercellular molecular exchange can be followed *in vivo* by fluorescence recovery after photobleaching (FRAP) analysis with fluorescent markers such as calcein, 5-carboxyfluorescein, or the sucrose analog esculin (14–16). Transfer appears to occur by simple diffusion, suggesting the existence of direct connections between the cytoplasm of adjacent cells in the filament (14, 16, 17).

## SEPTAL PROTEINS

Genes encoding septal proteins were originally identified in *Anabaena* by the inability of their mutants to grow diazotrophically (18–20). Inactivation of the *sepJ* gene (also known as *fraG*) hampers heterocyst differentiation at an early step and produces a strong filament fragmentation phenotype both in the presence and absence of combined nitrogen, albeit stronger in the latter conditions (18, 19). Inactivation of genes in the *fraCDE* operon produces filament fragmentation in the absence of combined nitrogen, and their mutants form heterocysts that are impaired in nitrogen fixation mainly under oxic conditions (15, 20). The proteins encoded by all these genes have membrane-embedded domains, and, as viewed with fusions to the green fluorescent protein (GFP), SepJ, FraC, and FraD are localized to the intercellular septa along the filaments of *Anabaena* (18, 20); this location has been corroborated for FraD and SepJ by immunolocalization with specific antibodies (15, 21). Notably, SepJ is focused in the center, whereas FraD is more spread in the intercellular septa.

The *sepJ* and *fraC-fraD* mutants of *Anabaena* are impaired in intercellular molecular exchange determined by FRAP analysis (14–16), but their phenotypes are not identical. Thus, the *fraC-fraD* mutant is more impaired in 5-carboxyfluorescein and esculin transfer than the *sepJ* mutant (15, 16), whereas impairment in calcein transfer is more similar in the *sepJ*, *fraC*, *fraD*, and double *fraC-fraD* mutants (14, 16, 20). Additionally, the *sepJ* and *fraC-fraD* mutants are impaired in the formation of septal disk nanopores but to different extents: in the *sepJ* mutant, about 18% (16) or 42% (12) of the wild-type number of nanopores remain, whereas in the *fraC-fraD* mutant, about 14%–17% of the wild-type number remain (12, 16). A triple *sepJ fraC-fraD* mutant contains the lowest number of nanopores, 8%–10% of the wild-type number (12, 16). Significantly, a strict correlation between calcein transfer and nanopore number is observed in filaments incubated in the absence of combined nitrogen (in the presence of nitrogen, regulatory effects appear to affect the correlation; 12). On the other hand, conventional TEM with potassium permanganate staining shows the presence of connecting structures in the intercellular septa of the filaments (see, e.g., Wilk et al. and Flores et al. [11, 22]). All these observations led to the proposal that the septal proteins constitute proteinaceous *septal junctions* (23) that connect the cytoplasm of adjacent cells in the filament traversing the septal PG through the nanopores (16). Further, two types of septal junctions were proposed to exist: those related to SepJ and those related to FraCD (15, 16).

## SEPTAL JUNCTIONS VISUALIZED

A major step toward the understanding of septal junctions was the study of the intercellular septa in *Anabaena* filaments by cryo-electron tomography (cryoET; 24). This study showed the presence of structures joining the adjacent cells that could be interpreted as septal junctions. They consisted of a tube traversing the septal PG that contained a plug and a cytoplasmic cap at each of its ends. Because the thickness of the septum is not homogenous being narrower at its center, the length of the tube varied to match the septum width at each location (24). Significantly, FraD could be identified as a component of the plug, and, consistent with previous results (15), FraC appeared to be necessary for FraD localization. The formation of these septal junctions was therefore impaired in *fraC* and *fraD* mutants, whereas they were apparently not altered in the *sepJ* mutant. Nonetheless, the shortest junctions observed in the wild type, which should correspond to those in the center of the septum, appear to be missing in the *sepJ* mutant (see Fig. S5A in Weiss et al. [24]).

Lack of identification of septal junctions specifically associated to SepJ has led to the proposal that this protein, rather than being a component of septal junctions, may have a general role in coordinating septal maturation (24) or as a dock initiating “channel” (junction) assembly (21). An important role of SepJ in septal maturation is consistent with the strong filament fragmentation phenotype of its mutants (discussed above); however, SepJ is evidently not necessary for the assembly of the FraD-containing septal junctions visualized by cryoET (24).

## PROTEIN INTERACTIONS

Besides SepJ and FraCD, a number of other septal proteins that interact with FraD or SepJ and influence filament integrity, intercellular molecular transfer, and the formation of nanopores have been identified in *Anabaena*. FraD-interacting protein SepN has been identified as a septal junction plug component, further defining the FraD-SepN junctions (25). On the other hand, SjcF1 is a PG-binding protein that has been shown to interact with SepJ in reconstitution of adenylate cyclase-based bacterial two-hybrid (BACTH) assays (26); the interaction may involve an SH3 domain in SjcF1 and an SH3-binding motif in SepJ (26). A *sjcF1* mutant, although not homozygous, is impaired in calcein transfer and exhibits a wider nanopore diameter than the wild type (29 ± 8.2 nm versus 21 ± 2.1 nm) (26). SepI is a protein that has coiled-coil and linker-like domains in a disposition similar to that of SepJ but lacks a permease domain (see SepJ structure below); a *sepI* mutant is affected in nanopore formation and in the intercellular transfer of calcein and 5-carboxyfluorescein, and the SepI protein interacts with SepJ in BACTH assays (27). SepT is another membrane-anchored, coiled-coil domain-containing protein that interacts with SepJ in BACTH assays and whose inactivation affects nanopore formation (28).

## PREDICTED SepJ STRUCTURE

**Fig 1:**
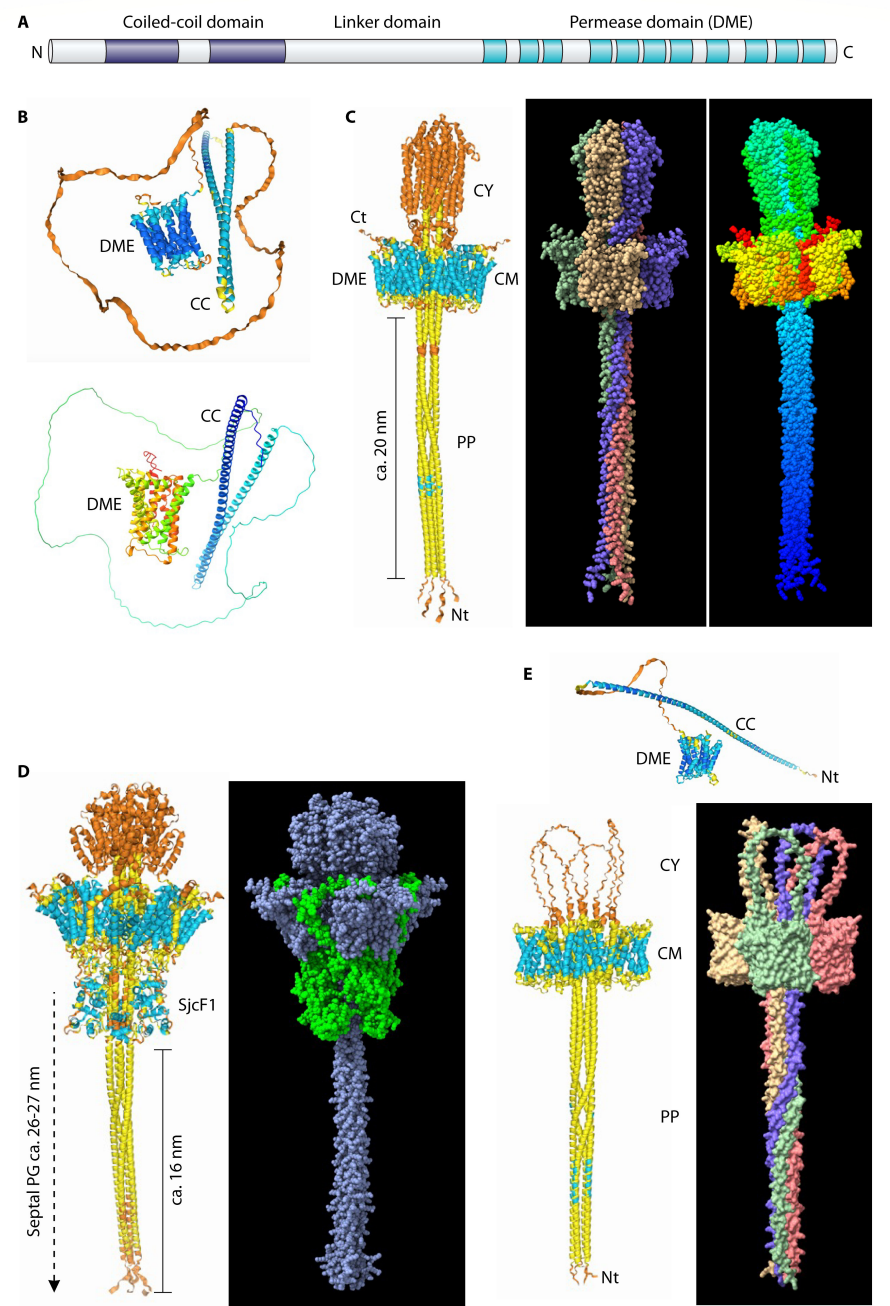
Structure of the SepJ protein. (A) Scheme of the SepJ protein from *Anabaena* sp. strain PCC 7120. Two coiled-coil structures are indicated as purple boxes, whereas transmembrane segments are indicated as turquoise boxes. (B) Structure of the SepJ monomer generated by AlphaFold 3 (*top*; blue to orange, high to low prediction probability) and the same structure visualized by ChimeraX (31) in rainbow representation (*bottom*; blue to red, N-terminal to C-terminal). The permease (DME) and coiled-coil (CC) structures are indicated. (C) Structure of a SepJ tetramer generated by AlphaFold 3 (*left*) and visualized by ChimeraX as atom-sphere representation, with each SepJ subunit in a different color (*middle*) or in rainbow representation (*right*). The predicted localization of various parts of the protein in different cellular compartments (CY, cytoplasm; CM, cytoplasmic membrane; PP, periplasm) is based on the known localization of the C-terminus (Ct) in a short cytoplasmic extension of the permease domain (18; see also Fig. S1B). Note the localization of the N-terminus (Nt) and part of the coiled-coil structure in the periplasm. (D) Joint structure of a SepJ tetramer and an SjcF1 tetramer generated by AlphaFold 3 (*left*) and visualized by ChimeraX (*right*) with the SepJ (blue) and SjcF1 (green) tetramers in different colors. SjcF1 is a CM-anchored protein with two periplasmic PG-binding domains (26). Septal PG is about 26 to 27-nm-thick (see the text). (E) SepJ protein from *Pseudanabaena* sp. strain PCC 7367. The monomer structure generated by AlphaFold 3 (*top*) contains strongly predicted permease (DME) and coiled-coil (CC) structures. The tetramer structure (*bottom*) generated by AlphaFold 3 (*left*) and visualized by ChimeraX (*right*) with each subunit in a different color is similar to that of *Anabaena* SepJ but with a shorter linker domain. The position of the N-terminus is indicated (Nt).

SepJ consists of coiled-coil, linker, and integral membrane (permease) domains (Fig. 1A; described in detail in Herrero et al. [2]). The coiled-coil domain consists of two distinct coiled-coil structures; the linker domain has a high proportion of Pro, Ser, and Thr residues; and the integral membrane domain is homologous to proteins in the drug and metabolite exporter (DME) family, a member of the drug and metabolite transporter superfamily (TCDB superfamily 2.A.7; https://tcdb.org). Prediction of the structure of the *Anabaena* SepJ monomer by AlphaFold 3 shows well-structured coiled-coil and integral membrane domains and an unstructured linker domain (Fig. 1B). SepJ shows strong self-interactions in BACTH assays (29), and it has been isolated from *Anabaena* as a multimeric protein, mostly as a tetramer, although other possible SepJ-containing complexes could also be detected (30). AlphaFold 3 was then asked to model an *Anabaena* SepJ tetramer. A structure showing the coiled-coil domains within or passing through the center of a four-permease complex was obtained in 92% of 25 predictions, with the N-terminus and different extensions of the coiled-coil domain located in the periplasm in 64% of the predictions, whereas the bulk of the linker domain was most frequently (92% of the predictions) located in the cytoplasm (see one representative prediction in Fig. 1C; a few other predictions are shown in Fig. S1, including a prediction of a SepJ-GFP fusion protein [18] and another one highlighting the coiled-coil and permease domains and a view of the protein from the periplasm). The fact that different lengths of the coiled-coil domain can be observed in the periplasm (Fig. S1A) suggests that no strong interactions between the coiled-coils and the permease tetramer are detected to fix a structure. However, adding four subunits of the PG- and SepJ-binding protein SjcF1 to the model consistently resulted in a SepJ structure with the N-terminus and a long stretch of the coiled-coil domain located in the periplasm and SjcF1 located in a position likely allowing its interactions with PG (10 out of 10 predictions; see an example in Fig. 1D; adding two SjcF1 monomers instead of four had the same effect in three out of five predictions). SepJ and SjcF1 can have extensive interactions involving their CM and periplasmic domains (Fig. S1D).

The structure of the long (204 amino acids) linker domain of *Anabaena* SepJ is predicted with low probability. SepJ is widely found in filamentous cyanobacteria (32), with the coiled-coil and permease domains being strongly conserved, whereas the linker domain is very variable in length and sequence, although conserved in amino acid composition (2). Therefore, the structure of SepJ from *Pseudanabaena* sp. PCC 7367, a filamentous cyanobacterium phylogenetically distant from *Anabaena* that has a short (72 amino acids) linker domain, was tested. The structure predicted is similar to that of *Anabaena* SepJ, with the N-terminus and a substantial part of the coiled-coil domain located in the periplasm (five predictions were run with similar results; see one representative prediction in Fig. 1E). The linker domain, although much shorter, is located in the cytoplasm as most frequently predicted for *Anabaena* SepJ. It can be suggested that a function for the linker domain is positioning of the coiled-coil domain to pass through the center of the permease tetramer.

To test whether the SepJ structure predicted for *Anabaena* and *Pseudanabaena* sp. PCC 7367 could be widespread in filamentous cyanobacteria, AlphaFold 3 was used to predict the structure of SepJ tetramers from other 18 filamentous cyanobacteria, 10 heterocyst-formers and eight non-heterocyst formers (three or four predictions for each protein). Overall, 65% of those additional predictions located the coiled-coil domain through the permease tetramer center, and in 62% of these, the N-terminus was located in the periplasm (Table S1). Interestingly, in addition to *Anabaena* and *Pseudanabaena* sp. PCC 7367, this structure was predicted at least once for the SepJ protein of eight out of 10 heterocyst formers and of five out of eight non-heterocyst formers. Thus, the structure of the SepJ tetramer in which the coiled-coil domains traverse the permease domain complex locating the N-termini in the periplasm may be found in many filamentous cyanobacteria. Nonetheless, further work would be necessary to confirm each structure, and it remains possible that some cyanobacteria produce a SepJ protein that conforms to a non-canonical structure.

## PERIPLASMIC SepJ COILED-COIL DOMAIN

The periplasmic location of the coiled-coil domain of *Anabaena* SepJ has been questioned because the protein lacks any evident Sec or twin-arginine translocation (TAT) signal peptide for secretion (21). However, the AlphaFold 3-predicted SepJ structure raises the possibility of a different mechanism to allocate the coiled-coil domain in the periplasm, not necessarily involving the Sec or TAT machineries. Further, the presence of the coiled-coil domain in the periplasm is supported by experimental observations. Thus, in addition to the interaction with ScjF1 discussed above, both the complete SepJ protein and its isolated coiled-coil domain have been shown to interact with PG preparations from *Anabaena* in pull-down assays (30).

When two monomers of SepI were added to SepJ, AlphaFold 3 predicted structures in which SepI was well integrated into the SepJ complex, with large areas of contact between SepI and SepJ involving both the coiled-coil domains and the linker (SepJ) or linker-like (SepI) domains (Fig. S2). It is possible that interaction between the linker/linker-like domains of the two proteins helps to locate the coiled-coil domains of SepI together with those of SepJ through the SepJ permease complex and in the periplasm. A SepT dimer could also be integrated into the SepJ complex, with its transmembrane domains inserted between the permeases of SepJ and the coiled-coil domains in the periplasm; extensive interactions between the transmembrane domains and between the periplasmic coiled-coil domains of the two proteins are predicted (Fig. S3). (In the AlphaFold 3 predictions, just one SepI or SepT monomer was hardly integrated in the SepJ tetramer, and more than two SepI or SepT monomers were also not integrated in the SepJ tetramer.) These structural predictions offer a hypothetical molecular basis for the described interactions of SepI and SepT with SepJ (27, 28).

The filament fragmentation phenotype of a *sepJ* mutant is nullified by inactivation of *amiC1* encoding a cell wall hydrolase, N-acetylmuramoyl-L-alanine amidase (33), which has been involved in drilling the nanopores (13, 34). This suggests that SepJ has a direct or indirect role as an inhibitor of AmiC1 and that in the absence of SepJ, AmiC1 splits septal PG resulting in filament fragmentation. The periplasmic location of the SepJ coiled-coil domain is of interest because a Phyre2 structural homology search (35) identifies, among the structures most similar to the SepJ coiled-coil domain, periplasmic coiled-coil domains involved in inhibition of bacterial cell wall hydrolases such as the coiled-coil domain of a RipA-type *Corynebacterium glutamicum* PG hydrolase (36). In this context, in AmiC of *Escherichia coli*, an α-helix has been shown to bind and inhibit its own catalytic domain (37). An inhibitory α-helix is however absent from cyanobacterial AmiC proteins, as shown for AmiC2 from *Nostoc punctiforme* (38) that is globally similar to *Anabaena* AmiC1. To explore the idea of a hypothetical inhibition by SepJ of the PG-splitting activity of AmiC1, the possible interaction of the SepJ coiled-coil domain and the catalytic domain of AmiC1 was examined with AlphaFold 3. Different predictions showed different sequences of the SepJ coiled-coil domain that could be involved in such interaction, and, therefore, further work will be necessary to define a candidate interacting sequence. Nonetheless, these observations raise the possibility that SepJ is a direct inhibitor of the AmiC1 amidase in *Anabaena.*

## SepJ-RELATED NANOPORES

The possible location of the SepJ coiled-coil domain in the periplasm raises the question of the relation of SepJ with the septal PG. The coiled-coil domain of SepJ, comprising two consecutive coiled-coil structures, has a predicted extension of about 30 nm (see Fig. S1C), which is similar to the size of the septal periplasm, with a mean distance between the adjacent cells of about 38 nm that gets narrower, 25–30 nm, at the center (24). It is therefore possible that, together with other interacting proteins such as SepI or SepT and in an extended conformation into the periplasm, the coiled-coil domain of SepJ traverses the septal PG.

As described above, a *sepJ* mutant shows a decreased number of nanopores (18%–42% of the wild-type number in different studies), whereas an *Anabaena* SepJ-overexpressing strain shows an about 1.4-fold increase in the number of nanopores (39). The nanopores that remain in a *fraC-fraD* mutant occupy a central position in the septal disks, in contrast to the more spread position of the nanopores in the septal disks of the wild-type or of the *sepJ* mutant (Fig. S4). Altogether, these observations suggest that, in addition to the nanopores associated to the FraD-SepN junctions, there are nanopores specifically associated to the SepJ protein, which is more focused at the center of the septum than the FraC or FraD proteins (20, 39). On the other hand, the nanopores that remain in the triple *sepJ fraC-fraD* mutant may be associated to the cap- and plug-lacking tubes of the FraD-SepN junctions as suggested by Weiss et al. (24).

## THE SepJ PERMEASE DOMAIN

As mentioned earlier, the SepJ permease domain is strongly conserved, at least in heterocyst-forming cyanobacteria (2), and is homologous to proteins in the DME family. DME permeases show broad substrate specificity, and members of this family can transport substrates such as methyl viologen or a number of different amino acids (40). SepJ has been shown to specifically affect the intercellular transfer of *patS-* and *hetN-*related morphogens (39, 41). Additionally, overexpression of SepJ specifically increases transfer of the fluorescent marker calcein from vegetative cells to heterocysts (39). Mutations of some amino acids predicted to be positioned close to or in the cytoplasmic face of the permease domain of SepJ specifically affect the intercellular transfer of calcein (42). In the AlphaFold 3-predicted structure for SepJ, these amino acids indeed occupy a position facing the cytoplasm (Fig. S5), suggesting that they may be involved in the access of calcein (and, hence, of other substrates) to the SepJ permease. It therefore can be hypothesized that, in addition to holding the coiled-coil domains as a tetramer, the highly conserved SepJ permease has a role in molecular transfer across the CM.

## FUTURE PROSPECTS

Localization of SepJ to the intercellular septa is dependent on the divisome (29), and numerous interactions between divisome proteins and SepJ have been described in *Anabaena* (43). However, because the divisome is dismantled when cell division is complete whereas SepJ remains at the center of the septum, interactions between divisome proteins and SepJ are likely transient. Additionally, a complex relation of SepJ to sugar transporters that are required for full intercellular calcein transfer and, in one case, nanopore formation and SepJ localization at the intercellular septa has been described (44). On the other hand, SepJ is required for full activity of an ABC transporter for acidic amino acids to be manifest (45). These observations emphasize the integration of SepJ with other proteins affecting septal maturation and the physiology of filamentous cyanobacteria.

The interactions of the SepJ tetramer with proteins such as SjcF1, SepI, or SepT can account for some SepJ-containing complexes larger than the SepJ tetramer that have been observed in SepJ preparations from *Anabaena* (30). Whether some of those complexes could involve several different proteins in addition to SepJ remains to be determined, and further work would be necessary to explore their possible structures. Additionally, we have previously hypothesized that SepJ from adjacent cells could interact with each other through their coiled-coil domains forming a cell-cell connecting structure (18). Whether interaction of SepJ from adjacent cells actually occurs and how the SepJ complexes could contribute to make a structure mediating the intercellular transfer of substrates (including morphogens) is unknown. However, the AlphaFold 3-predicted structure of SepJ tetramers with a periplasmic coiled-coil domain makes it plausible the interaction of SepJ from adjacent cells and should stimulate the search for such intercellular structures.

## SUPPLEMENTAL MATERIAL

Supplemental tables and figures jb.00488-24-s0001.pdf Table S1 and Figures S1 to S5.

ASM does not own the copyrights to Supplemental Material that may be linked to, or accessed through, an article. The authors have granted ASM a non-exclusive, world-wide license to publish the Supplemental Material files. Please contact the corresponding author directly for reuse.
