# Supplementary material for: The periplasmic and cytoplasmic faces of septal protein SepJ from filamentous cyanobacteria
Source: J Bacteriol. 2025 Mar 31;207(5):e00488-24. doi: 10.1128/jb.00488-24 (PMC12096826; doi:10.1128/jb.00488-24)
Supplement: Supplemental tables and figures — Table S1 and Figures S1 to S5. [file jb.00488-24-s0001.pdf]

Strain	SepJ length (amino acids)	Number of AlphaFold 3 predictions	Coiled-coils within permease tetramer	Periplasmic N-terminus
Heterocyst-formers				
Anabaena sp. PCC 7120 ❈	751	25	23	16
Anabaena sp. strain 90	751	3	1	1
Anabaena cylindrica PCC 7122	791	3	2	1
Calothrix sp. PCC 6303	628	3	2	1
Chlorogloeopsis sp. PCC 6912	633	4	2	0
Cylindrospermum sp. PCC 7417	847	3	3	3
Fischerella sp. PCC 6905	690	3	2	2
Mastigocladopsis sp. PCC 10914	647	4	3	0
Nostoc sp. PCC 7524	747	3	3	2
Nostoc azollae	728	3	3	1
Nostoc punctiforme ATCC 29133	779	3	1	1
Non-heterocyst-formers				
Pseudanabaena sp. PCC 7367 ❈	565	5	5	5
Arthrospira sp. NIES-39	566	4	2	0
Geitlerinema sp. PCC 7407	602	3	2	1
Oscillatoria sp. PCC 10802	627	3	3	3
Nodosilinea sp. PCC 7104	627	3	2	2
Phormidium sp. HE10JO	579	4	2	0
Prochlorothrix sp. PCC 9006	624	4	0	0
Pseudanabaena sp. PCC 7429	584	3	3	3
Trichodesmium sp. IMSI101	583	4	3	3

Suppl. Table S1. Filamentous cyanobacteria for which the structure of SepJ tetramers was predicted using AlphaFold 3. SepJ length, number of predictions run, number of times the coiled-coils were positioned traversing the central cavity of the permease tetramer, and number of times the N-terminus was located in the periplasm are indicated. The asterisks denote strains whose SepJ tetramer is specifically discussed in the text.

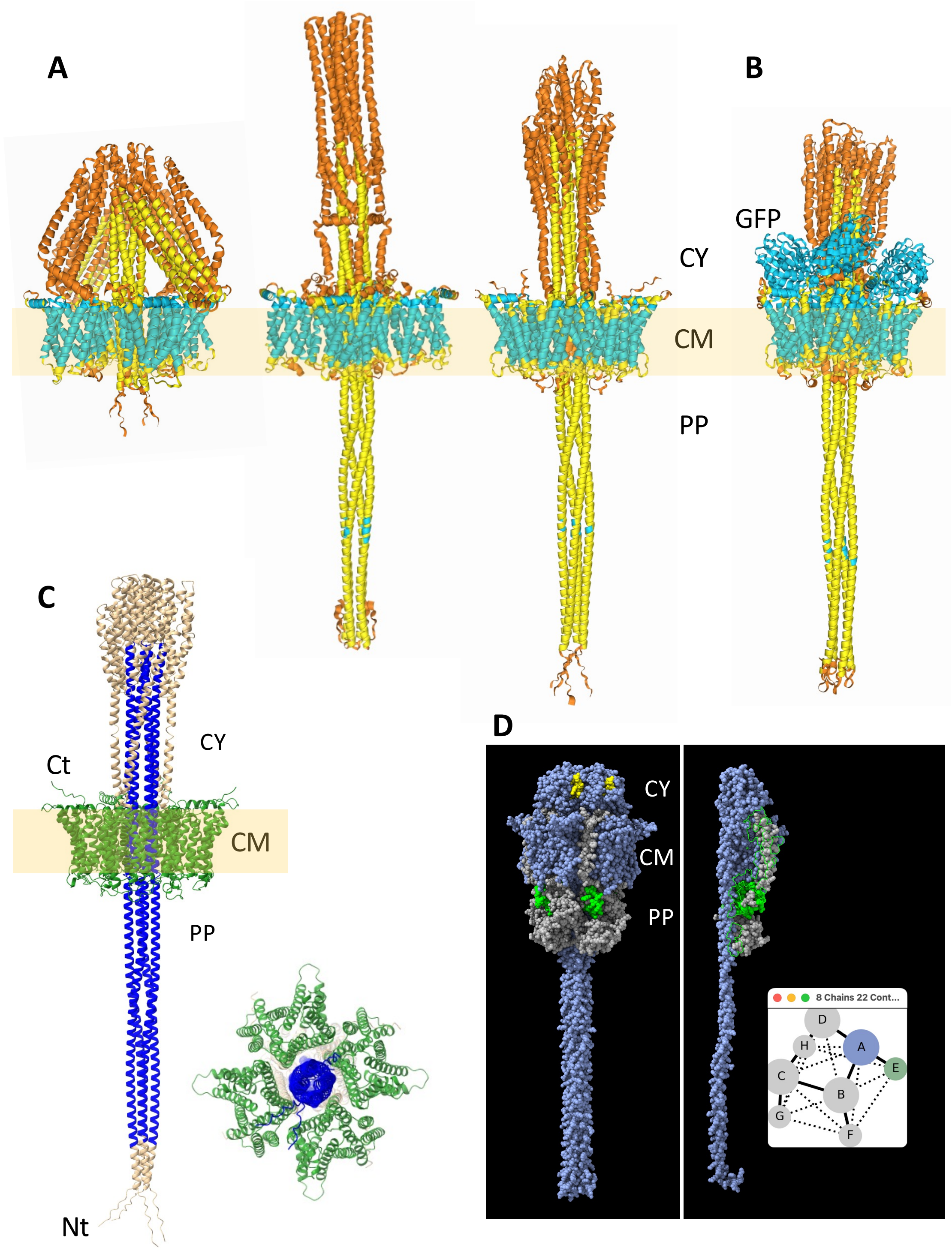

Suppl. Figure S1. Alternative structures of SepJ from *Anabaena* sp. strain PCC 7120. (A) Three different AlphaFold 3 SepJ tetramer-predicted structures showing different lengths of the coiled-coil domain into the periplasm. (B) Predicted AlphaFold 3 structure of the SepJ-GFP protein of strain CSAM137 (1). Note that the GFP, added close to the C-terminus of SepJ, is located in the cytoplasm as originally suggested from the observed functionality of the GFP used, GFPmut2, which is fluorescent only in the cytoplasm. (C) (*Left*) An AlphaFold 3-predicted SepJ tetramer with the conserved coiled-coil domain highlighted in blue and the transmembrane (DME permease) domain in green (colored with ChimeraX); in this conformation, the coiled-coil domain has a length of about 30 nm. (*Right*) The SepJ tetramer viewed from the periplasm (amino acid residues 1-207, blue; DME permease, green). (D) (*Left*) Complex of four SepJ (blue) and four SjcF1 (grey) proteins showing the SH3 domain of SjcF1 (lime) and the SH3-binding motif of SepJ (yellow), which are in distant locations in the mature complex suggesting that their possible interaction (2) may be transient during the formation of the complex. (*Right*) Some polypeptides were removed from the complex to show as an example the interactions (highlighted by a green line) between SepJ polypeptide A and SjcF1 polypeptide E (the inset summarizes contact interfaces in the complex; large circles, SepJ monomers; small circles, SjcF1 monomers; solid or dotted lines, larger or smaller interfaces, respectively). CY, cytoplasm; CM, cytoplasmic membrane (cream color); PP, periplasm; Ct, C-terminus; Nt, N-terminus.

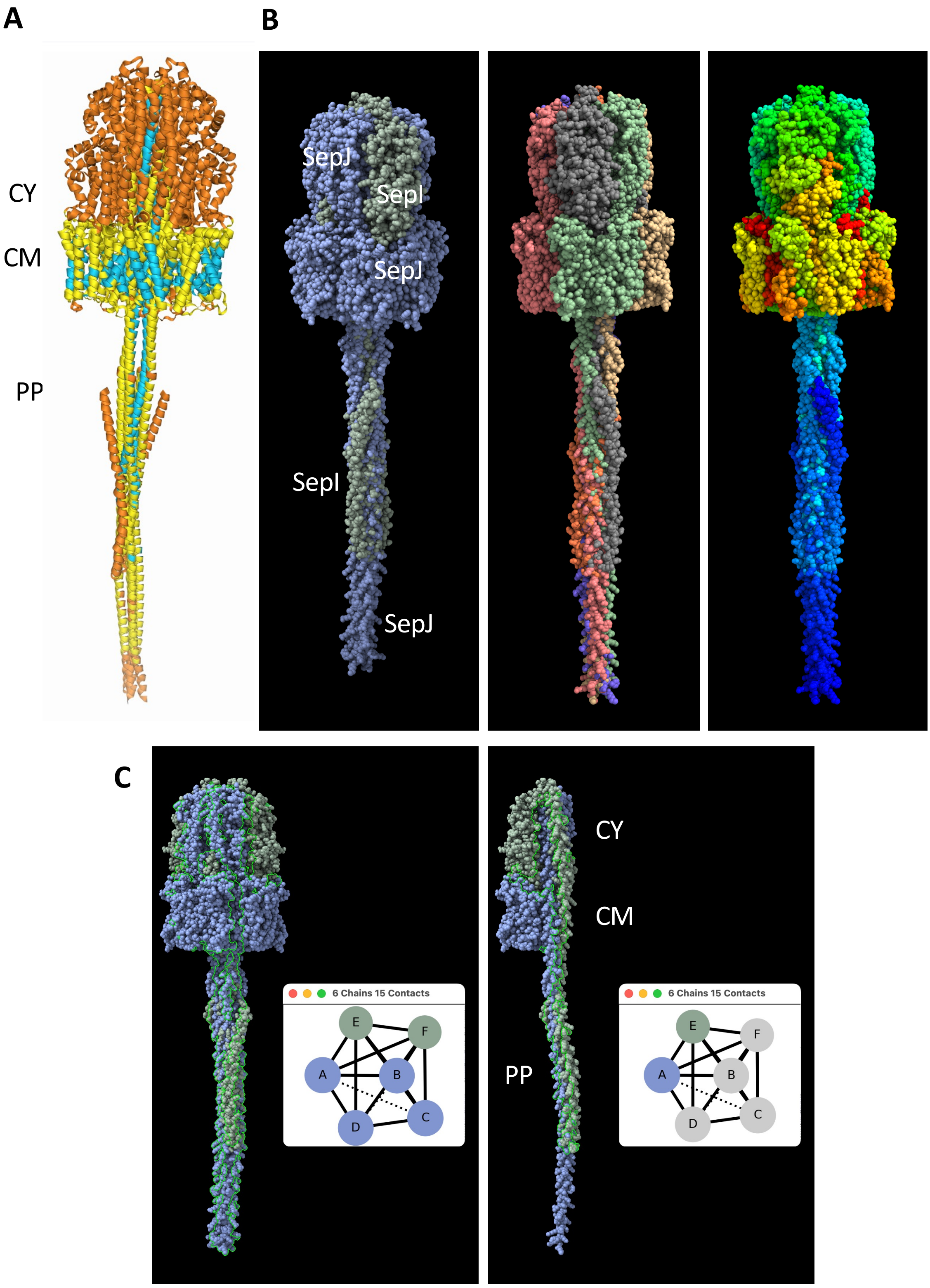

Suppl. Figure S2. *Anabaena* SepJ-SepI complex. (A) AlphaFold 3 predicted structure for a SepJ tetramer-SepI dimer showing that the two proteins are well integrated into a single complex. (B) ChimeraX views of the complex: SepJ shown in blue and SepI in olive green (*left*), each polypeptide shown in a different color (*middle*; SepI monomers in orange and grey, respectively), and rainbow representation (*right*). The structure predicts that, like in SepJ, most of the SepI coiled-coil domain is located in the periplasm and the linker-like domain in the cytoplasm (see also C, *right*). (C) (*Left*) Extensive contacts (highlighted by a green line) are detected between the polypeptides of the complex (inset: A-D, SepJ; E-F, SepI); (*right*) some polypeptides were removed to show as an example the interactions between SepJ polypeptide A and SepI polypeptide E. CY, cytoplasm; CM, cytoplasmic membrane; PP, periplasm.

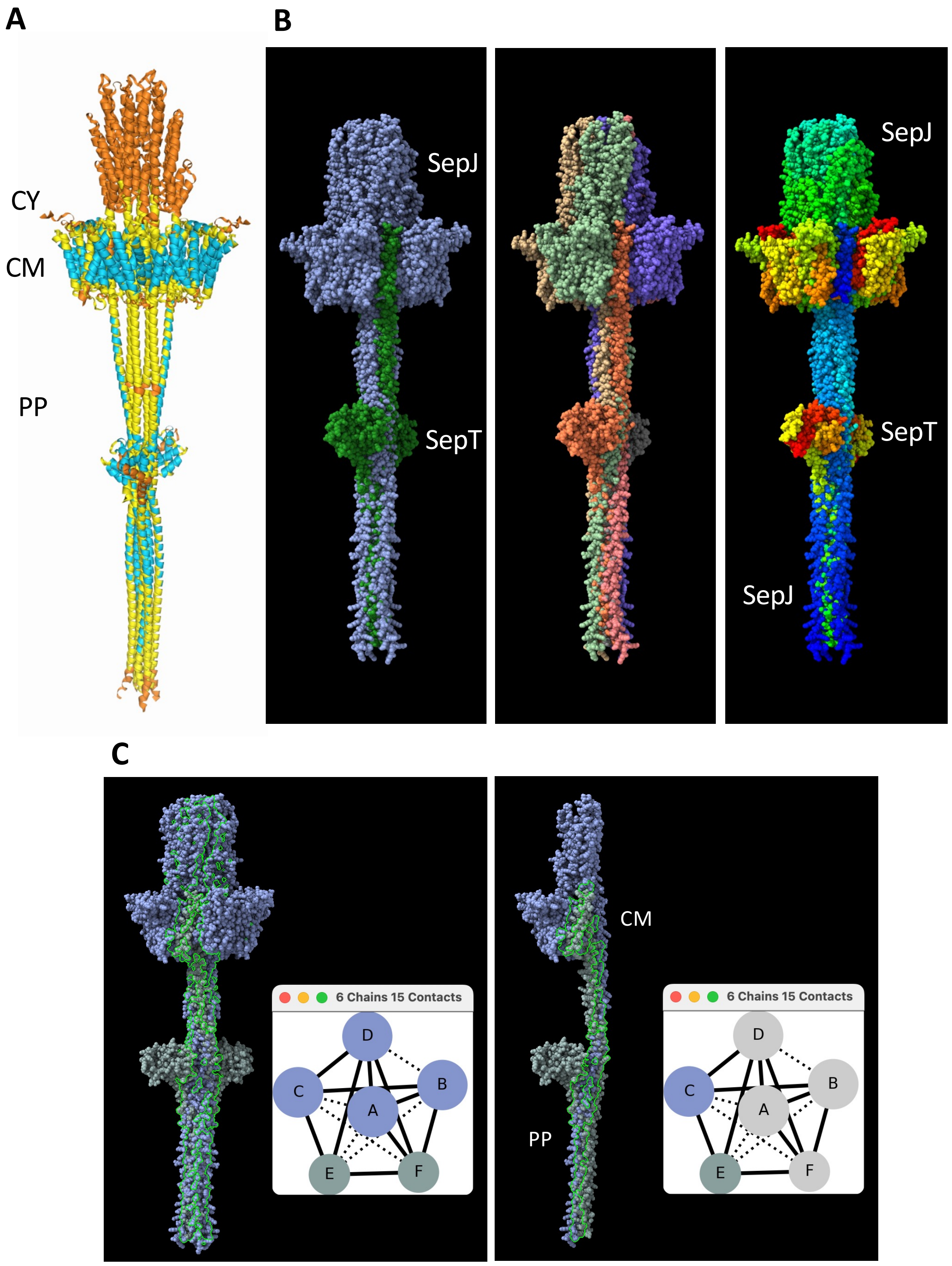

Suppl. Figure S3. *Anabaena* SepJ-SepT complex. (A) AlphaFold 3 predicted structure for a SepJ tetramer-SepT dimer showing that the two proteins can be integrated into a single complex. (B) ChimeraX views of the complex: SepJ shown in blue and SepT in green (*left*), each polypeptide shown in a different color (*middle*; SepT monomers in orange and grey, respectively), and rainbow representation (*right*). The structure predicts that the SepT coiled-coil domain is located in the periplasm and its transmembrane domains in the cytoplasmic membrane. (C) (*Left*) Extensive contacts (highlighted by a green line) are detected between the polypeptides of the complex (inset: A-D, SepJ; E-F, SepT); (*right*) some polypeptides were removed to show as an example the interactions between SepJ polypeptide C and SepT polypeptide E. CY, cytoplasm; CM, cytoplasmic membrane; PP, periplasm.

A PCC 7120 CSVM34 (Δ*sepJ*) CSVT22 (Δ*fra*Δ*fraD*)

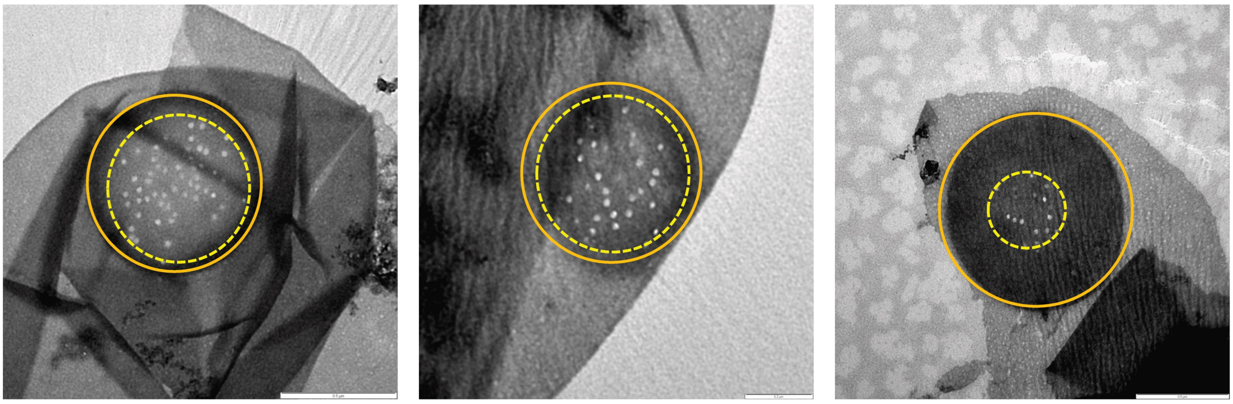

	Anabaena sp. PCC 7120 (n = 13)		CSVM34 (n = 17)		CSVT22 (n = 9)	
**Diameter**	**Disk**	**nnp zone**	**Disk**	**nnp zone**	**Disk**	**nnp zone**
**Mean (µm)**	0.788	0.460	0.742	0.485	0.933	0.337
**SD (µm)**	0.171	0.093	0.231	0.094	0.162	0.072

B **Heterocyst preparations** **Mostly vegetative cells**

PCC 7120 CSVT22 (Δ*fra*Δ*fraD*) PCC 7120 CSVT22 (Δ*fra*Δ*fraD*)

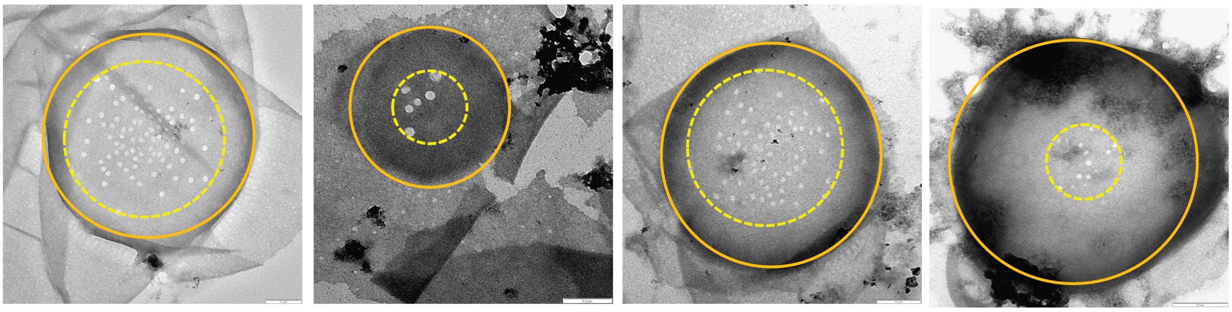

	**Heterocyst preparations**				**Mostly vegetative cells**			
	Anabaena sp. PCC 7120 (n = 7)		CSVT22 (n = 7)		*Anabaena* sp. PCC 7120 (n = 14)		CSVT22 (n = 9)	
**Diameter**	**Disk**	**nnp zone**	**Disk**	**nnp zone**	**Disk**	**nnp zone**	**Disk**	**nnp zone**
**Mean (µm)**	1.046	0.763	0.856	0.343	1.341	0.811	1.347	0.407
**SD (µm)**	0.103	0.101	0.289	0.117	0.104	0.164	0.206	0.056

Suppl. Figure S4. Localization of nanopores in septal peptidoglycan disks of *Anabaena* sp. PCC 7120 (WT) and mutants CSVM34 (Δ*sepJ*) and CSVT22 (Δ*fraC* Δ*fraD*). Peptidoglycan was isolated (A) from filaments grown in nitrate-containing BG11 medium (shaken cultures; 3), which consist mostly of vegetative cells, or (B) from heterocyst-enriched or non-enriched preparations of filaments grown in combined N-free BG11$_0$C medium (bubbled cultures) (4); the different preparations were visualized by electron microscopy (3, 4). The area of the septal disks (orange circles) was determined for the number of disks indicated (n), and in each disk the zone occupied by nanopores was delimited (yellow dotted circles) and its area was determined (nnp zone). Disk size was not significantly different between the WT and any of the mutants under any of the conditions tested (Student's *t* test $P \geq 0.059$). Nanopore zone size was not different in mutant CSVM34 and the WT ($P = 0.477$ [BG11 medium]), whereas it was significantly smaller in mutant CSVT22 than in the WT in the three sample types studied ($P \leq 0.0023$). In BG11-grown filaments (A), the nnp zone occupied on average 34% (WT), 43% (Δ*sepJ*) and 13% (Δ*fraC* Δ*fraD*) of the disk area. In BG11$_0$C-grown filaments (B), the nnp zone occupied on average 53% (WT) and 16% (Δ*fraC* Δ*fraD*) of the disk area in heterocyst disks, and 37% (WT) and 9% (Δ*fraC* Δ*fraD*) in vegetative cell disks (the Δ*sepJ* mutant could not be studied because it does not grow in medium lacking combined nitrogen). Thus, the nanopores remaining in strain CSVT22 (Δ*fraC* Δ*fraD*) occupy a smaller area than the nanopores in the WT in the three samples (on average, area in CSVT22/area in WT, about 1/3), whereas the nanopores remaining in strain CSVM34 (Δ*sepJ*) occupy an area not smaller than that in the WT in the conditions investigated (BG11 medium). (The original micrographs are from the studies published in refs. 3, 4.)

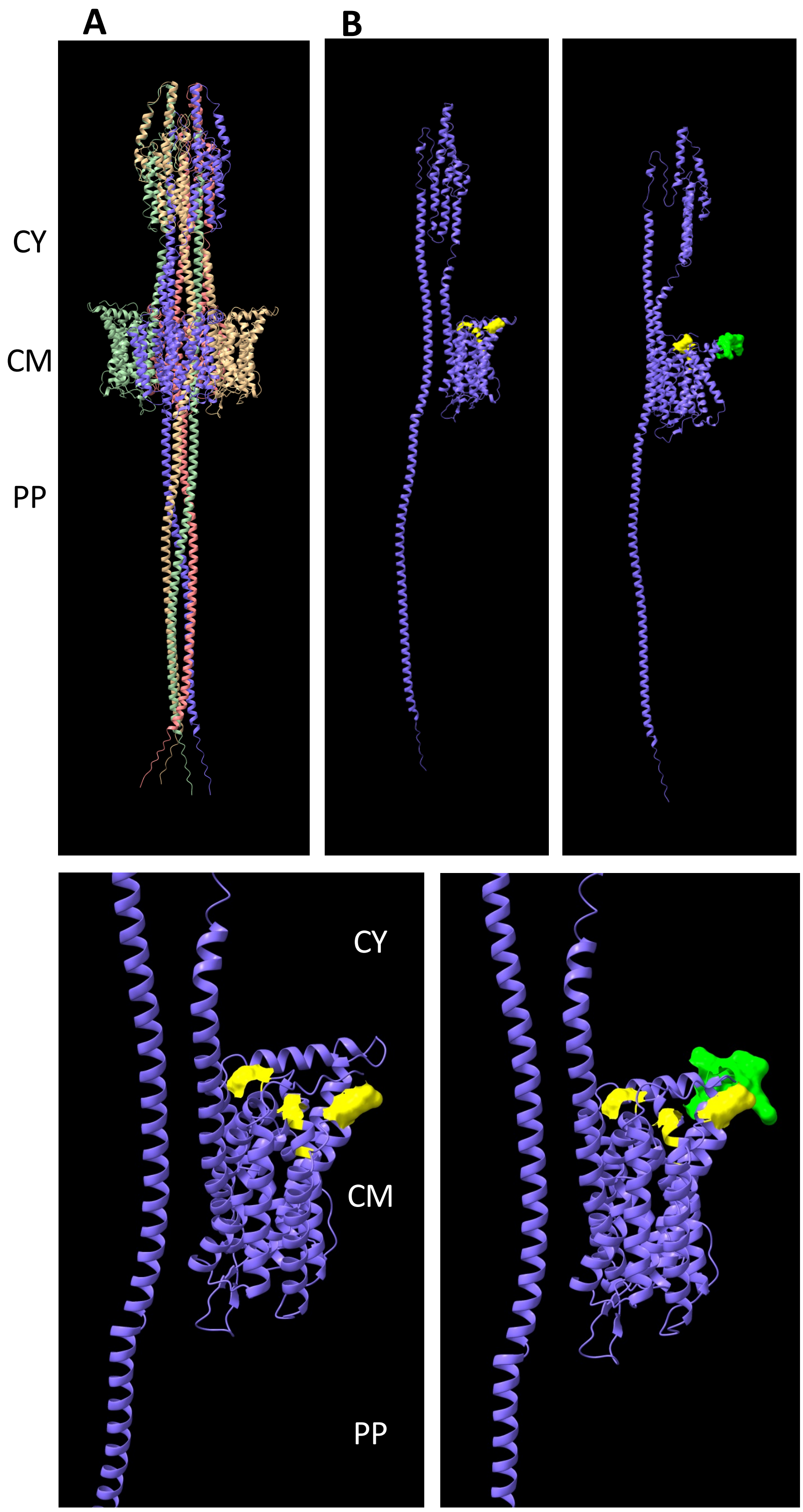

Suppl. Figure S5. *Anabaena* SepJ permease mutants affected in intercellular calcein transfer. (A) ChimeraX view of an AlphaFold 3 predicted structure for a SepJ tetramer, each polypeptide in a different color (ribbon representation). (B) To facilitate visualization, three polypeptides were removed from the complex, and mutations whose major effect is to impair intercellular calcein transfer (5) were located: R562A, Y612A, T616A, R617A, H624A (yellow color), and a L498 to S507 deletion (green color). Lower images are magnifications of the corresponding top images. Approximate location of cellular compartments are indicated: CY, cytoplasm; CM, cytoplasmic membrane; PP, periplasm.

Supplemental material references

1. Flores E, Pernil R, Muro-Pastor AM, Mariscal V, Maldener I, Lechno-Yossef S, Fan Q, Wolk CP, Herrero A. 2007. Septum-localized protein required for filament integrity and diazotrophy in the heterocyst-forming cyanobacterium *Anabaena* sp. strain PCC 7120. J Bacteriol 189:3884-3890.

2. Rudolf M, Tetik N, Ramos-León F, Flinner N, Ngo G, Stevanovic M, Burnat M, Pernil R, Flores E, Schleiff E. 2015. The peptidoglycan-binding protein SjcF1 influences septal junction function and channel formation in the filamentous cyanobacterium *Anabaena*. mBio 6(4):e00376.

3. Arévalo S, Nenninger A, Nieves-Morión M, Herrero A, Mullineaux CW, Flores E. 2021. Coexistence of communicating and noncommunicating cells in the filamentous cyanobacterium *Anabaena*. mSphere 6(1):e01091-20.

4. Arévalo S, Flores E. 2021. Heterocyst septa contain large nanopores that are influenced by the Fra proteins in the filamentous cyanobacterium *Anabaena* sp. strain PCC 7120. J Bacteriol 203(13):e0008121.

5. Ramos-León F, Arévalo S, Mariscal V, Flores E. 2018. Specific mutations in the permease domain of septal protein SepJ differentially affect functions related to multicellularity in the filamentous cyanobacterium *Anabaena*. Microb Cell 5(12):555-565.
